# Synthesis, Characterization and Structure Properties of Biobased Hybrid Copolymers Consisting of Polydiene and Polypeptide Segments

**DOI:** 10.3390/polym13213818

**Published:** 2021-11-04

**Authors:** Nikolaos Politakos, Ioannis Moutsios, Gkreti-Maria Manesi, Dimitrios Moschovas, Ainur F. Abukaev, Evgeniia A. Nikitina, Galder Kortaberria, Dimitri A. Ivanov, Apostolos Avgeropoulos

**Affiliations:** 1 Department of Materials Science Engineering, University of Ioannina, 45110 Ioannina, Greece; nikolaos.politakos@ehu.eus (N.P.); imoutsios@uoi.gr (I.M.); gretimanesi@uoi.gr (G.-M.M.); dmoschov@uoi.gr (D.M.); 2 POLYMAT and Departamento de Química Aplicada, Facultad de Ciencias Químicas, University of the Basque Country UPV/EHU, Joxe Mari Korta Zentroa, Tolosa Etorbidea 72, 20018 San Sebastián, Spain; 3 Faculty of Chemistry, Lomonosov Moscow State University (MSU), GSP-1, 1-3 Leninskiye Gory, 119991 Moscow, Russia; abukaev.af@phystech.edu (A.F.A.); nikitina.ea@phystech.edu (E.A.N.); dimitri.ivanov@uha.fr (D.A.I.); 4 Institute of Problems of Chemical Physics, Russian Academy of Sciences, 142432 Moscow, Russia; 5 ‘Materials + Tecnologies’ Research Group, Chemistry and Environmental Engineering Department, Faculty of Engineering, University of the Basque Country (UPV/EHU), Plaza Europa 1, 20018 Donostia, Spain; galder.cortaberria@ehu.es; 6 Institut de Sciences des Matériaux de Mulhouse–IS2M, CNRS UMR7361, 15 Jean Starcky, 68057 Mulhouse, France

**Keywords:** biobased hybrid copolymers, polyisoprene, polybutadiene, polypeptide, anionic polymerization, molecular characterization, thermal properties, fibril morphology

## Abstract

Novel hybrid materials of the PB-*b*-P(o-Bn-L-Tyr) and PI-*b*-P(o-Bn-L-Tyr) type (where PB: 1,4/1,2-poly(butadiene), PI: 3,4/1,2/1,4-poly(isoprene) and P(o-Bn-L-Tyr): poly(ortho-benzyl-L-tyrosine)) were synthesized through anionic and ring-opening polymerization under high-vacuum techniques. All final materials were molecularly characterized through infrared spectroscopy (IR) and proton and carbon nuclear magnetic resonance (^1^H-NMR, ^13^C-NMR) in order to confirm the successful synthesis and the polydiene microstructure content. The stereochemical behavior of secondary structures (α-helices and β-sheets) of the polypeptide segments combined with the different polydiene microstructures was also studied. The influence of the α-helices and β-sheets, as well as the polydiene chain conformations on the thermal properties (glass transition temperatures, thermal stability, α- and β-relaxation) of the present biobased hybrid copolymers, was investigated through differential scanning calorimetry (DSC), thermogravimetric analysis (TGA) and dielectric spectroscopy (DS). The obtained morphologies in thin films for all the synthesized materials via atomic force microscopy (AFM) indicated the formation of polypeptide fibrils in the polydiene matrix.

## 1. Introduction

Copolymers constitute a broad class of materials that has been extensively studied in various scientific fields ranging from nanotechnology [1] up to drug delivery systems [2,3]. On the other hand, polypeptides have recently attracted scientific interest due to their unique thermal, mechanical and self-assembly properties [4,5]. By combining the remarkable structural variety and functionality of polypeptides with the exquisite properties of synthetic polymers, novel biobased hybrid materials can be obtained [3].

The stiff α-helices of the polypeptide segments lead to rod–coil conformations of the final hybrid materials [6,7]. The asymmetry of the stiff rod may possibly induce chemical immiscibility between the two segments consisting of synthetic polymer chains and polypeptide segments, resulting in a higher Flory–Huggins interaction parameter (*χ*) [8]. The aforementioned class of materials has been investigated in the literature, and the combination of synthetic-biological segments has been referred to as “molecular chimeras” [9,10].

Peptide-based hybrid copolymers consisting of purely synthetic hydrocarbon chains and biopolymers adopt interesting self-assembled structures [11,12]. It should be noted that well-defined supramolecular structures, i.e., nanofibers, as well as micellar morphologies and solid-state assemblies, have enabled the use of rod–coil copolymers in numerous applications related to biocompatibility, resistance to enzymatic degradation and recognition binding (active targeting), refs. [13,14,15] drug delivery, biomedicine, biosensors and biomineralization [16,17,18,19,20]. In addition, membrane-, refs. [21,22,23,24,25] micelle-, ref. [26] hydrogel-, ref. [13] electronic- [27] and coating-related [28] applications have been proposed.

In order to synthesize hybrid materials consisting of one polydienic (polybutadiene or polyisoprene) or vinyl block with various peptides, different synthetic approaches have been proposed in the literature [7,8,9]. Specifically, synthetic protocols such as ring-opening polymerization of amino acid-N-carboxy-anhydrides (NCA), metal initiators [24], amine hydrochloride salts [25], chain-transfer agents [29,30,31,32] and the preparation of a functional macroinitiator baring amino groups able to initiate the polymerization of a peptide [10] have been reported.

Employing the aforementioned procedures a variety of hybrid materials has been synthesized combining synthetic segments (polystyrene, poly(butadiene), poly(isoprene), poly(dimethylsiloxane), poly(ethylene glycol), poly(ethylene) and poly(methyl methacrylate)) with biocompatible blocks such as poly(γ-benzyl-L-glutamate), poly(lysine), poly(phenylalanine), poly(alanine), poly(glycine), etc. [10].

The substantial progress on complex architecture hybrid systems utilizing living anionic and/or ring-opening polymerization techniques [6,26,33,34,35] has led to well-defined topologies due to the conformational asymmetry deriving from the α-helices and β-sheets of the rod-like segment. Finally, it should be noted that the use of different polymerization techniques (atom transfer radical polymerization, reversible addition-fragmentation chain transfer, ring-opening polymerization and nitroxide-mediated polymerization) has enabled the synthesis of hybrid materials for biological applications [13,36,37].

Herein, we report the synthesis, molecular and thermal characterization and self-assembly behavior of the polydienic/polypeptide diblock copolymers of the poly(isoprene)-*b*-poly(o-benzyl-L-tyrosine) or PI-*b*-P(o-Bn-L-Tyr) and the poly(butadiene)-*b*-poly(o-benzyl-L-tyrosine) or PB-*b*-P(o-Bn-L-Tyr) type. The protected L-tyrosine segment was preferred in order to avoid possible side reactions during the polymerization procedure due to the hydroxyl (-OH) groups in the monomeric unit of the peptide. Size exclusion chromatography (SEC) was used in order to calculate the total number average molecular weight of each segment, as well as the dispersity indices of all the final hybrid materials. Regarding the verification of the polydiene microstructures and the successful synthesis, proton nuclear magnetic resonance ^1^H-NMR was utilized. ^13^C-NMR was also employed for the interpretation of the chemical structure of the polypeptide, since the existence of various hydrogen atoms in the monomeric unit of P(o-Bn-L-Tyr) renders the justification of the secondary structures rather challenging. Infrared spectroscopy (IR) was used to further analyze all the characteristic chemical groups of both polydiene and polypeptide segments, as well as to evaluate the secondary structures of the polypeptide block. Through differential scanning calorimetry (DSC) and thermogravimetric analysis (TGA) the glass transition temperature of both segments, as well as the mixing temperature of the corresponding blocks, was calculated. Dielectric spectroscopy (DS) was employed in order to identify the α- and β-relaxation temperatures of the P(o-Bn-L-Tyr) block, as well as the possible differentiations to polydienic microstructures. Finally, the structural behavior of the hybrid materials in the thin-film state was studied with atomic force microscopy (AFM), since the polydiene segments enable the solubility and processability in common organic solvents.

## 2. Materials and Methods

### 2.1. Materials

1,3-Butadiene (99+%), isoprene (≥99%), sec-BuLi (1.4 M in cyclohexane), 1,2-dipiperidinoethane (dipip, 98%) hexane (≥97%) and tetrahydrofuran (THF, 99.9%) were purified according to the standards required for anionic polymerization and high-vacuum techniques [38,39]. Dipip was used as a polar reagent in order to promote a high 1,2 microstructure for the PB segment and -3,4/1,2 for the PI for the polydienic chains. In this study, the amino acid used was o-Bn-L-tyrosine (99%), and it was submitted to prepolymerization modification in order to receive the corresponding N-carboxy anhydride (NCA) according to the literature [30]. The purification of the NCA was accomplished through the recrystallization process using a solvent/nonsolvent system (THF/hexane) [31]. Triphosgene (99%) was used as received under vacuum in order to synthesize the NCA. The initiator 1,6-hexamethylenediamine (99%) was purified through sublimation and subsequently diluted in purified N,N-dimethylformamide (DMF, 99.8%). The procedure has already been thoroughly reported elsewhere [40,41,42]. Phosgene in toluene solution (20%) was used to substitute the lithium end group to chloro-substituted PB and PI segments, enabling the reaction with the hexamethylenediamine, in order to obtain the macroinitiator for the polymerization of the NCA protected tyrosine. All reagents were purchased from Sigma-Aldrich (St. Louis, MO, USA).

### 2.2. Methods

*SEC:* Molecular characterization was accomplished using a Perkin-Elmer (Waltham MA, USA) chromatograph equipped with a binary pump and a refractive index (RI) detector. The eluent used was THF, and separation was carried out with four columns packed with particle gels with different nominal pore sizes. The elution rate was set at 1 mL/min at 35 °C. The molecular weights were calculated based on a calibration curve from monodisperse polystyrene standards.

*FTIR:* Infrared spectroscopy (FTIR) was performed with a Nicolet Nexus 670 spectrometer (Wake Forest, NC, USA) equipped with a single horizontal golden gate attenuated total reflectance (ATR) cell. Spectra were recorded by averaging 20 scans between 4000 and 400 cm^−1^ with a resolution of 2 cm^−1^.

*^1^H-NMR and ^13^C-NMR:* Samples were dissolved in deuterated chloroform (CDCl_3_). The spectra were acquired at room temperature on an Avance Bruker 500 MHz (Rheinstetten, Germany) equipped with a BBO z-gradient probe Bruker DSX NMR spectrometer using a rate of 5000 Hz, a frequency of 500 MHz and a delay of 1 s.

*DSC:* Experiments were conducted using a TA Instruments Q20 DSC instrument (TA Instruments Ltd., Leatherhead, UK). A heat rate of 5 °C/min was used under nitrogen atmosphere, where the first heating was carried out in order to eliminate the thermal history of all samples, followed by a cooling cycle, and finally, a second heating was employed and presented in the DSC section.

*TGA:* Thermogravimetric analysis was carried out by a Perkin-Elmer Pyris Diamond TG/DTA (Waltham, MA, USA). Samples of approximately 5 mg were heated with a rate of 5 °C/min from 25 °C to 700 °C, under inert atmosphere.

*AFM:* The surface morphology was studied using an atomic force microscope, Dimension 3100/Nanoscope IVA, Veeco from Digital Instruments (Plainview, NY, USA). The tapping mode was employed using an integrated silicon tip/cantilever (125 μm in length) at a scan rate of 1.0 Hz and a resonant frequency of ~300 kHz. The measurements were performed with 512 scan lines.

*DS:* Dielectric spectroscopy measurements were carried out by a Novocontrol Alpha high-resolution analyzer over a temperature range between −100 °C and 100 °C at a constant frequency of 1 kHz. The instrument is interfaced to a computer and equipped with a Novocontrol cryogenic system for temperature control. Samples were placed between the gold-plated electrodes in a sandwich configuration.

### 2.3. Synthesis of the Hybrid Material

Polydiene segments were synthesized using anionic polymerization under high-vacuum techniques. 1,3-Butadiene (0.07 mol) and/or isoprene (0.05 mol) were initiated by *sec*-BuLi (0.2 mmoles) in a nonpolar solvent (hexane), and the reaction was left to propagate for 24 h at room temperature. In order to receive high-1,2 microstructure for the PB and 3,4/1,2-PI blocks, the use of a polar agent was imperative; therefore, dipip was added to the solvent prior to the introduction of the initiator to alter the polarity. The ratio between sec-BuLi and dipip (0.4 mmoles) was 1:2 for PB and 1:3 (0.6 mmoles) for PI, respectively. In the presence of dipip, monomers were left to react for 24 h at 8 °C for better control of the polymerization reaction due to enhanced kinetics of anionic sites in the polar environment [39]. The number average molecular weights of all polydiene precursors were determined through SEC (Figure S1a), as presented in the Supporting Information and are given in Table 1.

**Figure 1:**
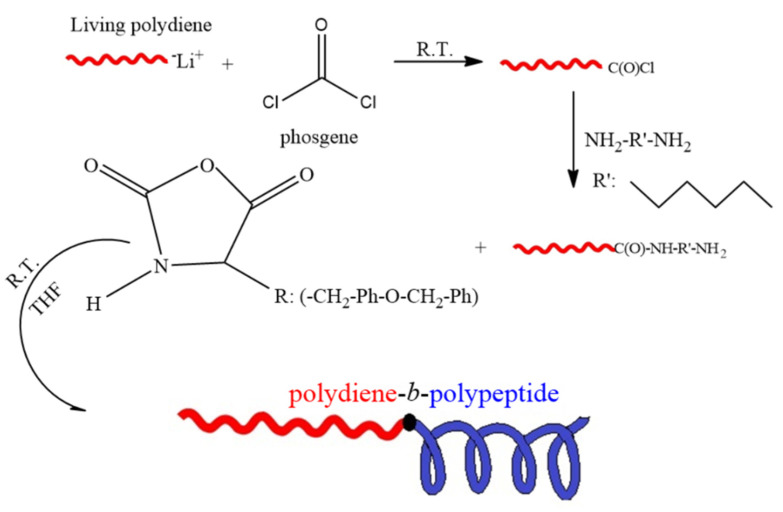
Schematic representation of the synthesis of the biobased hybrid materials.

After the complete polymerization of the polydiene blocks, the Li^+^ anionic centers were substituted to -Cl end groups able to initiate the polymerization of the NCA of the protected tyrosine using an excess of phosgene solution (5% in moles) for 24 h. Subsequently, the diamine solution was added in order to change the end group of the polydiene macroinitiators resulting in –NH_2_ side groups, and the reaction was completed after 24 h. The respective chromatographs of the functionalized polydiene precursors are presented in the Supporting Information (Figure S1b). Finally, the polymerization of the protected tyrosine was accomplished at room temperature after 24 h using THF as solvent. The hybrid materials were precipitated in methanol and dried under vacuum. The polymerization reaction in order to synthesize the coil–rod copolymer is presented in the Supporting Information (Figure S1c). In Figure 1 the synthetic route for the preparation of the biobased hybrid copolymers is presented.

## 3. Results and Discussion

### 3.1. Size Exclusion Chromatography

In order to verify the successful synthesis of all the hybrid materials, different characterization methods were employed. In total four samples were synthesized, specifically PI_1,4_-*b*-P(o-Bn-L-Tyr), PI_3,4/1,2_-*b*-P(o-Bn-L-Tyr), PB_1,4_-*b*-P(o-Bn-L-Tyr) and PB_1,2_-*b*-P(o-Bn-L-Tyr). For polydiene precursors, a small aliquot of the solution was retrieved in order to be molecularly characterized through SEC (Figure S1a). The synthetic route that followed for the preparation of the functionalized polydiene precursors ensured the substitution of a single chlorine atom. Vigorous stirring, temperature difference and specific glass apparatuses constituted the attributing factors for the successful synthesis of the functionalized precursors. After the substitution of the Li^+^ active sights with the respective Cl^-^ atoms of the phosgene reagent, an appropriate amount of diamine was introduced to the solution, resulting in –NH_2_ side groups. Small quantities of the functionalized precursors were also retrieved for characterization reasons by SEC (Figure S1b). The fact that the functionalized precursors showcase a slight increase in the dispersity but similar number average molecular weight (${\bar{M}}_{n}$) is attributed to the deviation of their hydrodynamic volume due to the chemical modification of the homopolymer with the respective –NH_2_ side groups. SEC chromatographs of the final hybrid materials are presented in the Supporting information (Figure S1c) and the molecular characteristics as directly calculated are presented in Table 1, where the relatively narrow dispersity indices (*Đ* < 1.1) indicate the molecular and compositional homogeneity.

### 3.2. Infrared Spectroscopy

**Figure 2:**
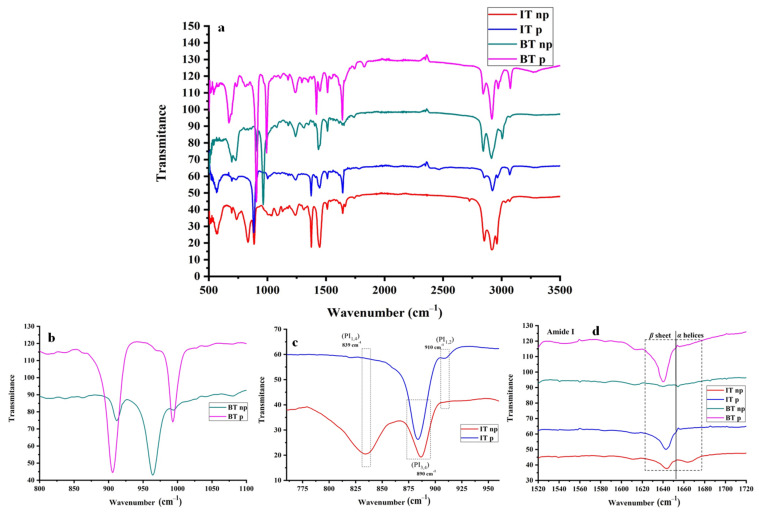
IR spectra correspond to all synthesized hybrid materials. The spectra in (**a**) correspond to wavelengths from 0 to 3500 cm^–1^, (**b**–**d**) to magnified areas from 800 to 1100 cm^−1^ and 760 to 950 cm^−1^ and 1520 to 1720 cm^−1^ respectively.

A powerful tool for the hybrid materials’ molecular analysis is IR spectroscopy, where the chemical groups of both polydiene and polypeptide chains can be precisely identified. Another benefit provided by the specific characterization method is the evaluation of secondary structures, since α-helices and β-sheets could be assigned to specific wavenumbers and random coil conformations. The secondary structures of the polypeptide chains could be determined through specific amide bands, where amide I is rendered more important and corresponds to stretching vibrations of the C=O bond of the peptide linkages. In addition, amide II derives from the in-plane -NH bending and from -CN stretching vibrations. Furthermore, amide III, corresponding to -CN stretching and -NH bending, amide IV to -OCN bending, amide V to out-of-plane -NH- bending and amide VI to out-of-plane C=O bending, can provide sufficient insight into the polypeptide secondary structures [43]. As far as the poly(butadiene) segments are concerned, different wavenumber values attributed to different PB microstructures are evident, specifically a strong band at 950 cm^−1^ for PB_1,4_ and at 900 cm^−1^, as well as 1000 cm^−1^ for PB_1,2_, respectively. Concerning the poly(isoprene) segments, the 1,4/3,4 microstructure are assigned to 839 cm^−1^ and 890 cm^−1^, respectively, while for the 1,2 microstructure at 910 cm^−1^. In Table S1, the transmittance wavenumber values of the respective polydiene chemical groups, as well as the amide groups of the polypeptide chains corresponding to the secondary structures, are presented. Furthermore, in Figure 2, the IR spectra for all samples are given, and the adjusted magnifications in the region of interest are presented separately for clarification reasons. Magnified spectra of amide III, IV, V and VI are presented in the Supporting Information (Figure S2a,b).

Specifically, for the case of PI_1,4_-*b*-P(o-Bn-L-Tyr), the PI microstructure consisting of 90% PI_1,4_ and 10% PI_3,4_, two distinctive peaks are evident at 839 cm^−1^ and 890 cm^−1^ (red-color spectrum), respectively, whereas in the case of PI_3,4/1,2_-*b*-P(o-Bn-L-Tyr), the absence of the 1,4 microstructure indicated that the reaction took place in a polar environment leading to 3,4/1,2 microstructures predominantly (blue-color spectrum) [44,45]. Coherent results were also acquired for the PB segment both in the case of nonpolar PB_1,4_-*b*-P(o-Bn-L-Tyr) and polar PB_1,2_-*b*-P(o-Bn-L-Tyr) depicted in green- and purple-color spectra in Figure 2, respectively.

The percentage of α-helices and β-sheets was also determined through the amide bands I and II, where the α-helices appear at approximately 1650 cm^−1^ (amide I) and 1540 cm^−1^ (amide II), while β-sheets emerge at 1625 cm^−1^ (amide I) and at 1520 cm^−1^ (amide II) [4,9,21,43,44]. Furthermore, the IR data revealed that for the cases of PI_3,4/1,2_-*b*-P(o-Bn-L-Tyr) and PB_1,2_-*b*-P(o-Bn-L-Tyr), secondary structures attributed to β-sheets can be clearly identified, but no α-helices are evident. Moreover, for PB_1,4_-*b*-P(o-Bn-L-Tyr), the bands shown could be attributed neither to α-helices nor β-sheets, while for PI_1,4_-*b*-P(o-Bn-L-Tyr), both α-helices or β-sheets are found with a ratio of 30 to 70%, respectively. In the case of tyrosine, and especially for the protected one, the helical conformation was not favorable due to the steric limitations deriving from the -R group, which is evident in the corresponding chemical structure (Figure 1) [46]. Finally, it is clear that steric effects arising from the polydiene polar microstructures favor sheet while prohibiting helical conformations.

### 3.3. Proton and Carbon Nuclear Magnetic Resonance

^1^H-NMR was mainly used for the determination of the microstructure of the polydiene segments, since the various hydrogen atoms in the polypeptide chains render the analysis rather insufficient. As a result, ^13^C-NMR was utilized in order to have a better clarification of the secondary structures of the polypeptides.

**Figure 3:**
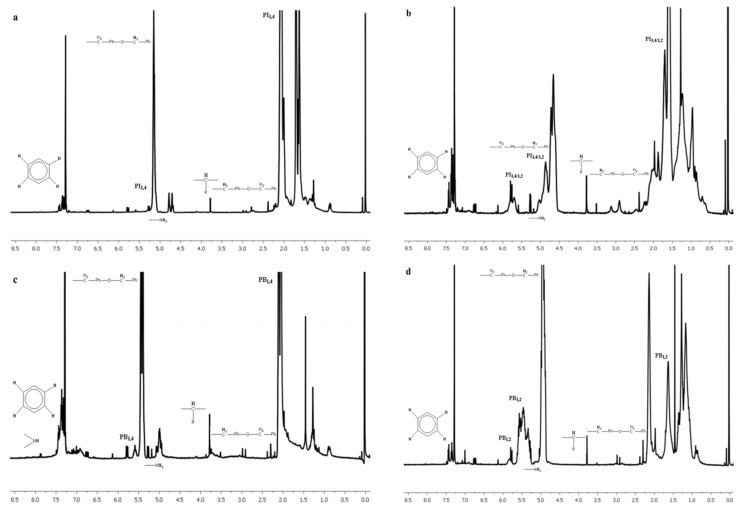
^1^H-NMR spectra of the synthesized materials corresponding to: (**a**) PI_1,4_-*b*-P(o-Bn-L-Tyr), (**b**) PI_3,4/1,2_-*b*-P(o-Bn-L-Tyr), (**c**) PB_1,4_-*b*-P(o-Bn-L-Tyr) and (**d**) PB_1,2_-*b*-P(o-Bn-L-Tyr).

In Table S2, the chemical shifts corresponding to the different chemical structures of all synthesized hybrid materials are summarized, further verifying the successful synthesis. In Figure 3, ^1^H-NMR spectra for the four synthesized samples are presented, indicating the different chemical shifts due to the various polydiene conformations, which is in accordance with the results obtained from the IR spectroscopy. The characteristic chemical shifts in the monomeric unit of both PB and PI have already been thoroughly reported in the literature and are in good agreement with the results presented in this study [44,45]. The -NH- groups in the main polypeptide chain are usually evident at 7.5 ppm [3,14], but due to the overlapping of the shift attributed to the deuterated solvent (CDCl_3_), a precise calculation of the contributing protons is not possible.

**Figure 4:**
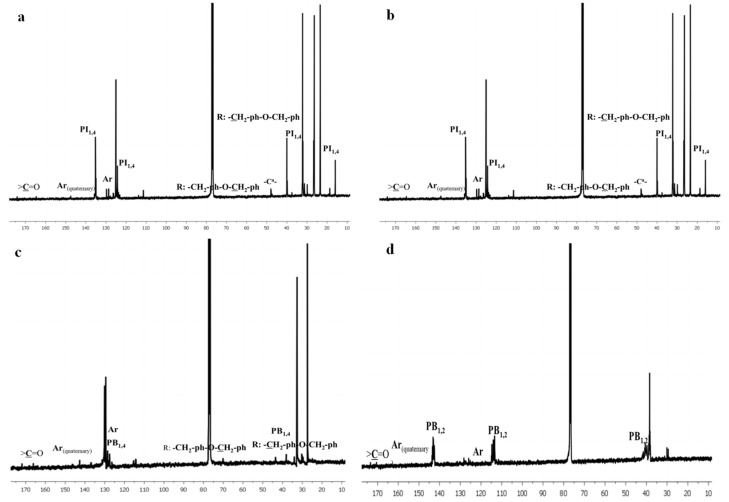
^13^C-NMR spectra of the synthesized materials corresponding to: (**a**) PI_1,4_-*b*-P(o-Bn-L-Tyr), (**b**) PI_3,4/1,2_-*b*-P(o-Bn-L-Tyr), (**c**) PB_1,4_-*b*-P(o-Bn-L-Tyr) and (**d**) PB_1,2_-*b*-P(o-Bn-L-Tyr).

For clarification and confirmation reasons, ^13^C-NMR experiments were also conducted, and the results are presented in Figure 4. The chemical shifts corresponding to the polydiene segments can be clearly identified in the theoretically expected regions, and the conjunction between the chemical groups and the corresponding chemical shifts is presented in Table S3.

Regarding the ITnp and ITp samples (Figure 4a,b), C^a^ of the main polypeptide chain is located at 55 ppm, confirming that the secondary structures are predominantly constituted by β-sheets since, according to the literature [47,48], C^a^ helical-type structures appear at 60 ppm. Furthermore, the sheet conformation was verified through the chemical shifts at 40 ppm that correspond to the first carbon of the R group (-CH_2_-ph-O-CH_2_-ph) [47,48]. The C=O linkage of the polypeptide chain at 174 ppm further supported the sheet conformation [47]. The tertiary aromatic carbons were found in the region 115–130 ppm, while the quaternary carbons appeared at 132 and 141 ppm, respectively. Finally, carbon atoms of the polydiene/diaminohexane and polydiene/polypeptide linkages were mainly observed beyond 165 ppm depending on the polydiene microstructures.

### 3.4. Thermogravimetric Analysis and Differential Scanning Calorimetry

The thermal stability of the hybrid materials was specified using thermogravimetric analysis. The thermal decomposition of the aforementioned materials can be strongly affected by the potential mixing of the components, as well as the secondary structures in the polypeptide chains. A higher degree of mixing is possible to lead to higher decomposition temperatures. Furthermore, secondary structures that bare hydrogen bonds among sheets and helices result in more thermally stable hybrid materials.

**Figure 5:**
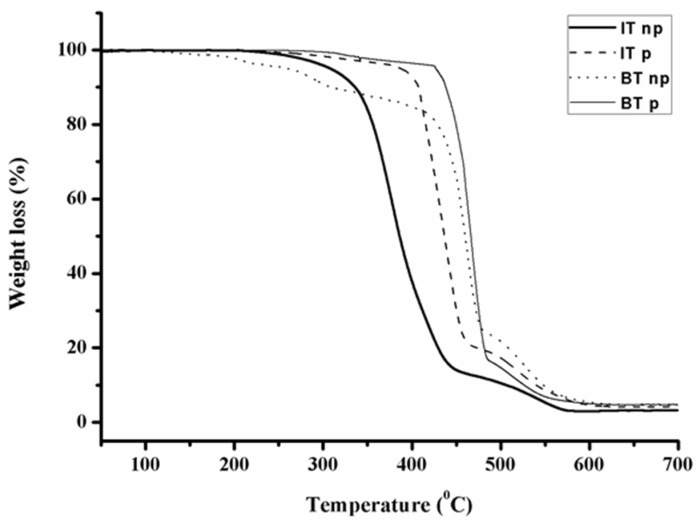
TGA thermographs of all the synthesized hybrid materials.

In Figure 5, the thermographs corresponding to the four different hybrid materials are presented with respect to the weight loss. For the diblock copolymer of the PB_1,2_-*b*-P(o-Bn-L-Tyr) type, it can be realized that the sample decomposes homogeneously at higher temperatures, while the secondary structures mainly consist of β-sheets. Overall, the hybrid materials decompose homogeneously at temperatures higher than 300 °C, except for the PB_1,4_-*b*-P(o-Bn-L-Tyr), in which the decomposition initiated at 250 °C but with a lower decomposition rate when compared to the rest of the hybrid materials. The higher thermal stability was evident for the cases of PI_3,4/1,2_-*b*-P(o-Bn-L-Tyr) and PB_1,2_-*b*-P(o-Bn-L-Tyr) due to the high percentage of the β-sheets of the polypeptide segment, as indicated by the IR spectra, as well as the existence of the more stable vinyl bonds along the main polydienic chains. Finally, after the complete decomposition of the polydiene segments, poly(o-Bn-L-Tyr) is decomposed homogenously for all samples, exhibiting a similar decomposition temperature, which is higher than 550 °C.

The glass transition temperatures (T*_g_*s) of the components, as well as the potential mixing between the chemically dissimilar blocks, were identified from DSC experiments. In the samples PI_3,4/1,2_-*b*-P(o-Bn-L-Tyr) and PB_1,2_-*b*-P(o-Bn-L-Tyr), the T*_g_*s are expected at temperatures equal to −30° for the PI_3,4_ (~55–60%) segment while for PB_1,2_ at approximately 0 °C, as already thoroughly reported in the literature [44,45,49,50]. The remaining polydienes, namely PI_1,4_ (in PI_1,4_-*b*-P(o-Bn-L-Tyr)) and PB_1,4_ )in PB_1,4_-*b*-P(o-Bn-L-Tyr)) display lower T*_g_*s, at approximately −70° and −90°, respectively [51,52,53].

**Figure 6:**
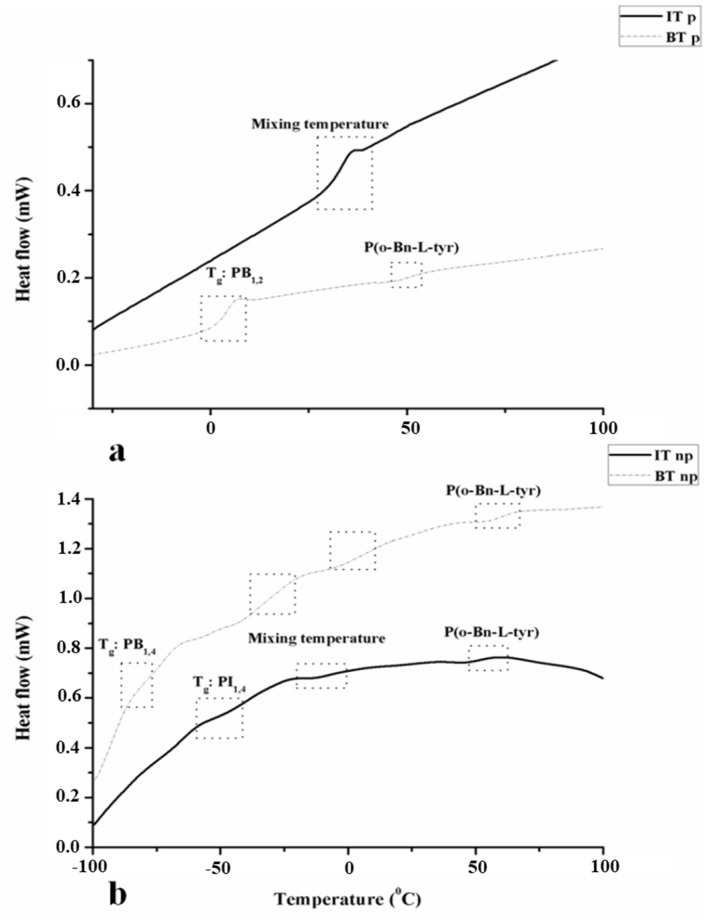
DSC thermographs of all synthesized hybrid materials are presented. In (**a**), the glass transition temperatures for both PI_1,4_-*b*-P(o-Bn-L-Tyr) (solid line) and PB_1,4_-*b*-P(o-Bn-L-Tyr) (dashed line) are obvious, as well as in (**b**) the glass transition temperatures for PI_3,4/1,2_-*b*-P(o-Bn-L-Tyr) (solid line) and PB_1,2_-*b*-P(o-Bn-L-Tyr) (dashed line).

In Figure 6, the DSC thermographs corresponding to the second heating of all four different samples are presented. Specifically, in Figure 6a, the thermographs of PI_3,4/1,2_-*b*-P(o-Bn-L-Tyr) (black solid line), where only one mixing T*_g_* at 33 °C was evident, indicate the miscibility of the specific system (leading to the lack of any nanostructure when studied morphologically). In addition, in the case of B_1,2_-*b*-P(o-Bn-L-Tyr) (gray dashed line), two different T*_g_*s were obvious, one at approximately 2 °C, corresponding to the PB_1,2_, and one at ~51 °C, attributed to the P(o-Bn-L-Tyr). As already presented in the IR spectra, the β-sheets evident in the PI_3,4/1,2_-*b*-P(o-Bn-L-Tyr) material may favor the mixing of the components. Furthermore, the total number average molecular weight of the PB_1,2_-*b*-P(o-Bn-L-Tyr) was higher than the PI_3,4/1,2_-*b*-P(o-Bn-L-Tyr). As a result, the mixing of the latter could be attributed to the lower molecular weight of the polypeptide block. In Figure 6b, thermographs corresponding to PB_1,4_-*b*-P(o-Bn-L-Tyr) and PI_1,4_-*b*-P(o-Bn-L-Tyr), two T*_g_* values close to the theoretically expected were evident (PB_1,4_: −80 °C and PI_1,4_: −55 °C), but also, two additional emerging T*_g_*s that deviated significantly from the respective polydiene homopolymers could be observed. These T*_g_*s resulted from the mixing of both PB or PI with the respective polypeptide in the two different samples (−27 °C for the PB_1,4_ and −10 °C PI_1,4_). For P(o-Bn-L-Tyr), the T*_g_*s were approximately evaluated at 60 °C and 53 °C, respectively. For the PB_1,4_-*b*-P(o-Bn-L-Tyr), the existence of two additional T*_g_*s (−27 °C and 3 °C) was attributed to the mixing of the components and the presence of vinyl poly(butadiene) (PB_1,2_~10%), which is obtained after polymerization in a nonpolar environment under anionic polymerization conditions and high-vacuum techniques [44,45].

### 3.5. Atomic Force Microscopy

**Figure 7:**
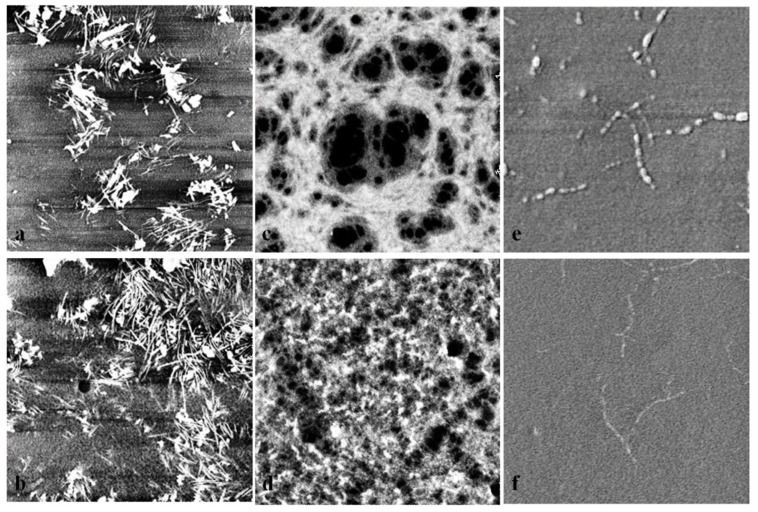
AFM images (3 μm × 3 μm) of PB_1,4_-*b*-P(o-Bn-L-Tyr) in room temperature and 80 °C (**a**,**b**, respectively), PB_1,2_-*b*-P(o-Bn-L-Tyr) in room temperature and 80 °C (**c**,**d**, respectively) and PI_1,4_-*b*-P(o-Bn-L-Tyr) in room temperature and 80 °C (**e**,**f**, respectively).

The surface morphology of the final biobased hybrid materials was studied with AFM in order to determine the self-assembly behavior of the chemically immiscible polypeptide and polydiene segments in thin films. All samples were cast onto glass substrates using the spin-casting method under specific conditions such as: spinning velocity ~3000 rpm for 30 s, polymer solution concentration equal to 3% wt in toluene, leading to film thickness of approximately 80 nm. The films were ex situ thermally annealed at 80 °C for 1 h (prior to the AFM investigation), a temperature higher than the T*_g_*s of all segments, as verified and discussed above, found from the DSC characterization. In Figure 7, the images of all samples except from PI_3,4/1,2_-*b*-P(o-Bn-L-Tyr) are presented. For sample PI_3,4/1,2_-*b*-P(o-Bn-L-Tyr), AFM imaging is not given, since no microphase separation was observed, as already verified by the respective DSC thermograph (only one T_g_ due to mixing was evident).

In the case of PB_1,4_-*b*-P(o-Bn-L-Tyr) the as-cast film (Figure 7a) exhibited fibril morphology (fibrils dimensions: 500 nm length and 30 nm width) with low aggregation degree and the annealed (Figure 7b) showcased more aggregated fibrils (fibrils dimensions: 1 μm length and 70 nm width), which is rather common for similar hybrid systems [53,54,55]. Fibril aggregations displaying different sizes ranging from micrometers down to a few nanometers have been reported in the literature and showed a direct relationship with a number of parameters such as polymer concentration, pH, secondary structures and chemical nature of the synthetic segment [55,56].

For the PB_1,2_-*b*-P(o-Bn-L-Tyr) sample, a network phase (Figure 7c) arising from the high immiscibility between the two components was observed. It should be mentioned that the secondary structures of the polypeptide chains hold a significant role in the formation of fibril morphology due to the presence of β-sheets. The secondary structures often assemble in parallel orientations and are capable of adopting tubular aggregations in similar systems [55]. The annealed sample (Figure 7d) exhibited a similar but more aggregated morphology due to the high percentage of β-sheets and the increased viscoelasticity of the PB segment.

Different results were obtained for the PI_1,4_-*b*-P(o-Bn-L-Tyr) sample where no significant microphase separation was observed during AFM studies, both at room temperature and at 80 °C. Sparsely disoriented fibrils were evident, probably due to the presence of secondary structures, α-helices and β-sheets (as given in IR spectra) that may prohibit the formation of any type of structure. In the Supporting Information (Figure S3), the 3D representation of the aforementioned sequences where the overlapping of the fibrils is clearly observed for the case of PB_1,4_-*b*-P(o-Bn-L-Tyr), the highly aggregated fibrils of the PB_1,2_-*b*-P(o-Bn-L-Tyr) hybrid material, as well as the poor dispersion of fibrils for the PI_1,4_-*b*-P(o-Bn-L-Tyr), are given.

### 3.6. Dielectric Spectroscopy

Dielectric spectroscopy was employed in order to recognize the different motional processes in all synthesized hybrid materials. A direct association with the obtained results from the DSC, regarding the various polydiene microstructures, is feasible, indicating segmental changes in the polypeptide related to secondary structures mixing with the polydienes and the α-relaxation. In general, polymeric systems at low temperatures behave as glassy solids, and an increase in temperature results in enhanced chain mobility and system flow. When rod–coil systems are employed, an additional parameter (the stiff backbone) must be taken into consideration.

**Figure 8:**
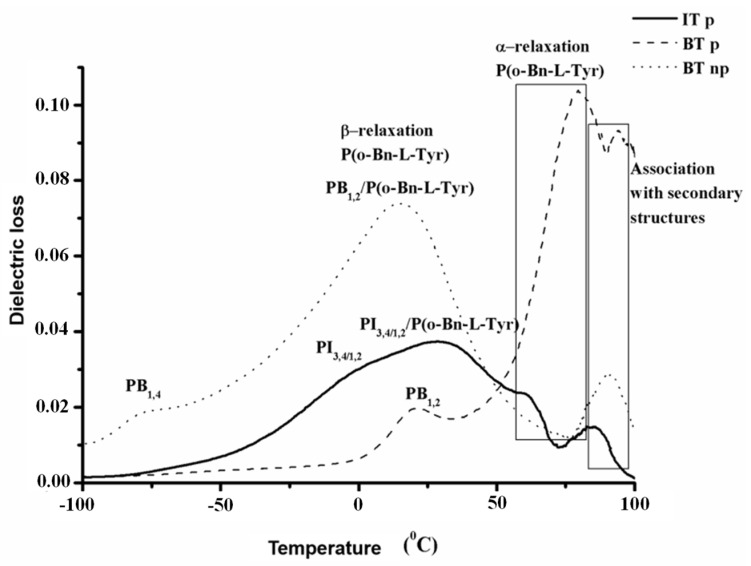
DS spectra of modulus vs. temperature for samples PI_1,4_-*b*-P(o-Bn-L-Tyr) (solid line), PB_1,4_-*b*-P(o-Bn-L-Tyr) (dotted line) and PB_1,2_-*b*-P(o-Bn-L-Tyr) (dashed line), respectively.

The double carbon bonds of the polydienic segments on the PI_1,4_-*b*-P(o-Bn-L-Tyr) and PB_1,4_-*b*-P(o-Bn-L-Tyr) samples are oriented parallel to the main chain (molecular dipole vectors), while in the cases of the PB_1,2_-*b*-P(o-Bn-L-Tyr) and PI_3,4/1,2_-*b*-P(o-Bn-L-Tyr) samples, the dipole moment is rigidly attached perpendicular to the hydrocarbon chain skeleton. In principle, secondary structures and α-relaxations are located at lower frequencies or higher temperatures than β-relaxation. The β-relaxation is strongly connected with the localized rotational fluctuations of the dipole vector, whilst the α-relaxation or dynamic glass transition is related to the calorimetric glass transition. It has been reported in the literature that the homopolypeptide of the protected tyrosine has a T_g_ value at 41 °C [46]. Dielectric measurements for the PB_1,4_ and PB_1,2_ segments indicated that the α-relaxation is located at −78 °C and 19 °C, while a secondary transition at −26 °C for the PB_1,2_ is attributed to the rotation of the side groups [57]. In Figure 8, the dielectric spectra for PB_1,2_-*b*-P(o-Bn-L-Tyr), PB_1,4_-*b*-P(o-Bn-L-Tyr) and PI_1,4_-*b*-P(o-Bn-L-Tyr), with respect to modulus and temperature are presented, clearly indicate that the different microstructures of the polydienic blocks have a strong effect upon the mixing between synthetic polymer/polypeptide segments.

For the PB_1,4_-*b*-P(o-Bn-L-Tyr) (dotted line), the intermediate process took place at 15 °C. The PI_3,4/1,2_-*b*-P(o-Bn-L-Tyr) (solid line) showcased the process at 28 °C and the PB_1,2_-*b*-P(o-Bn-L-Tyr) (dashed line) at 21 °C, which may be attributed to the segmental movement of the PB_1,2_ chains. Additionally, in the specific sample, the shape of the process is completely different compared to the other two samples. PB-containing samples exhibit pure processes, since the peak positions at −80 °C for the PB_1,4_ and 21 °C for the PB_1,2_ are in good agreement with the theoretically expected [57]. In the PI_3,4/1,2_-*b*-P(o-Bn-L-Tyr) sample, a respective peak position was not evident for the PI_1,4_; instead, a peak at −100 °C emerged, probably due to the rotation of the side groups [57]. As already described in the IR spectroscopy results in the PB_1,2_-*b*-P(o-Bn-L-Tyr) and PI_3,4/1,2_-*b*-P(o-Bn-L-Tyr) samples, the secondary structures evident in the polypeptide chains are predominately β-sheets, resulting in distinctive peak positions at 61 °C and 79 °C, respectively.

Finally, it should be mentioned that the processes related to the protected polytyrosine are found at higher values for the PB-containing samples, probably due to the chemical structure of the polybutadiene, which signifies an important role in the conformational changes.

## 4. Conclusions

In this work, the synthesis of novel biobased hybrid materials consisting of polydiene and polypeptide components using anionic and ring-opening polymerization under high-vacuum techniques was reported. In total, four copolymers presenting different geometric isomerisms for the polydienic segments, namely PI_1,4_-*b*-P(o-Bn-L-Tyr), PI_3,4/1,2_-*b*-P(o-Bn-L-Tyr), PB_1,4_-*b*-P(o-Bn-L-Tyr) and PB_1,2_-*b*-P(o-Bn-L-Tyr), were molecularly characterized through SEC, IR, ^1^H-NMR and ^13^C-NMR in order to verify the successful synthesis and to determine both the polydiene microstructures, as well as the secondary structures (α-helices and β-sheets) evident in the polypeptide chains. SEC chromatographs and ^1^H-NMR/^13^C-NMR spectra indicated the molecular and compositional homogeneity of all hybrid materials. Through IR spectroscopy, all wavenumber peaks corresponding to the characteristic chemical groups involved, as well as the existence of principally β-sheets in the polypeptide blocks, were documented. The thermal stability and the glass transition temperatures of all segments were specified through DSC and TGA experiments. It was found that the higher percentage of the polypeptide segments β-sheets, as well as stable vinyl bonds in the main polydienic chains, provided increased thermal stability. AFM experiments were conducted in order to study the phase behavior of the synthesized samples, where β-sheets, as secondary structures from the polypeptide block, combined with synthetic polydiene polymers can lead to fibril morphologies dispersed into the polymer matrix. Finally, through DS measurements, further analysis on the polydienic microstructures, as well as the a-relaxation, b-relaxation and secondary structures, was accomplished.

## Figures and Tables

**Table 1:** Total number average molecular weight and dispersity indices for polydiene and poly(o-Bn-L-tyr) segments corresponding to all samples as directly calculated from SEC.

Sample	Code Name	${\bar{M}}_{n}$ Polydiene (SEC) ^a^ g/mol	${\bar{M}}_{n}$ Polypeptide (SEC) ^a^ g/mol	${\bar{M}}_{n}$ (SEC) ^a^ g/mol	*Đ*_total_	f (%) Polypeptide
PI_1,4_-*b*-P(o-Bn-L-Tyr)	ITnp	14.300	41.500	55.800	1.10	74
PI_3,4/1,2_-*b*-P(o-Bn-L-Tyr)	Itp	16.400	44.200	60.600	1.08	73
PB_1,4_-*b*-P(o-Bn-L-Tyr)	BTp	15.400	59.500	74.900	1.09	79
PB_1,2_-*b*-P(o-Bn-L-Tyr)	BTnp	22.100	83.600	105.700	1.09	79

^a^ SEC was performed in THF at 35 °C.

## Data Availability

The data presented in this study are available upon request from the corresponding author.

## Supplementary Materials

The following are available online at https://www.mdpi.com/article/10.3390/polym13213818/s1, Figure S1: SEC chromatographs corresponding to: (a) polydiene precursors, (b) functionalized intermediate products and (c) all final biobased hybrid copolymers, Figure S2: Magnified spectra corresponding to amide: (a) III and (b) IV, V and VI, Table S1: Characteristic FTIR peak wavenumbers corresponding to all components of the synthesized hybrid materials, Table S2: Characteristic ^1^H-NMR chemical shifts corresponding to all components of the synthesized hybrid materials, Table S3: Characteristic ^13^C-NMR chemical shifts corresponding to all components of the synthesized hybrid materials, Figure S3: 3D AFM images (3 μm × 3 μm) at room temperature and the corresponding characteristic surface roughness profiles of (a) PB_1,4_-*b*-P(o-Bn-L-Tyr), (b) PB_1,2_-*b*-P(o-Bn-L-Tyr) and (c) PI_1,4_-*b*-P(o-Bn-L-Tyr).
