# Supplementary material for: Synthesis, Characterization and Structure Properties of Biobased Hybrid Copolymers Consisting of Polydiene and Polypeptide Segments
Source: Polymers (Basel). 2021 Nov 4;13(21):3818. doi: 10.3390/polym13213818 (PMC8588293; doi:10.3390/polym13213818)
Supplement: Supplementary file 1 [file polymers-13-03818-s001.zip › polymers-1440913-supplementary.pdf]

Synthesis, Characterization and Structure Properties of Biobased Hybrid Copolymers Consisting of Polydiene and Polypeptide Segments

Nikolaos Politakos[a,b], Ioannis Moutsios[a], Gkreti-Maria Manesi[a], Dimitrios Moschovas[a], Ainur F. Abukaev[c,d], Evgenia A. Nikitina[c,d], Galder Kortaberria[e], Dimitri A. Ivanov[c,d,f], Apostolos Avgeropoulos[a,c,*]

[a]Department of Materials Science Engineering, University of Ioannina, University Campus-Dourouti, 45110 Ioannina, Greece
[b]POLYMAT and Departamento de Química Aplicada, Facultad de Ciencias Químicas, University of the Basque Country UPV/EHU, Joxe Mari Korta zentroa, Tolosa etorbidea 72, Donostia-San Sebastián, 20018, Spain
[c]Faculty of Chemistry, Lomonosov Moscow State University (MSU), GSP-1, 1-3 Leninskiye Gory, 119991 Moscow, Russia
[d]Institute of Problems of Chemical Physics, Russian Academy of Sciences, Chernogolovka, 142432 Moscow, Russia
[e]'Materials+Tecnologies' Research Group, Chemistry and Environmental Engineering Department, Faculty of Engineering, Gipuzkoa, University of the Basque Country (UPV/EHU), Plaza Europa 1, 20018 Donostia, Spain
[f]Institut de Sciences des Matériaux de Mulhouse – IS2M, CNRS UMR7361, 15 Jean Starcky, Mulhouse 68057, France

***Corresponding author: Professor Apostolos Avgeropoulos**
E-mail address: aavger@uoi.gr

Supporting Information
The following data are given in the Supporting Information:
(A) SEC chromatographs of polydiene precursors, functionalized intermediate products and final biobased hybrid copolymers.
(B) Magnified spectra of amide III, IV, V, VI as well as IR characteristic wavenumbers for specific chemical groups of all hybrid materials (Table S1).
(C) ^{1}H-NMR & ^{13}C-NMR chemical shifts corresponding to all different chemical groups of the hybrid materials (Table S2, Table S3).
(D) 3D AFM images and the corresponding characteristic surface roughness profiles for $PB_{1,4}$-*b*-P(o-Bn-L-Tyr), $PB_{1,2}$-*b*-P(o-Bn-L-Tyr) and $PI_{1,4}$-*b*-P(o-Bn-L-Tyr) samples.

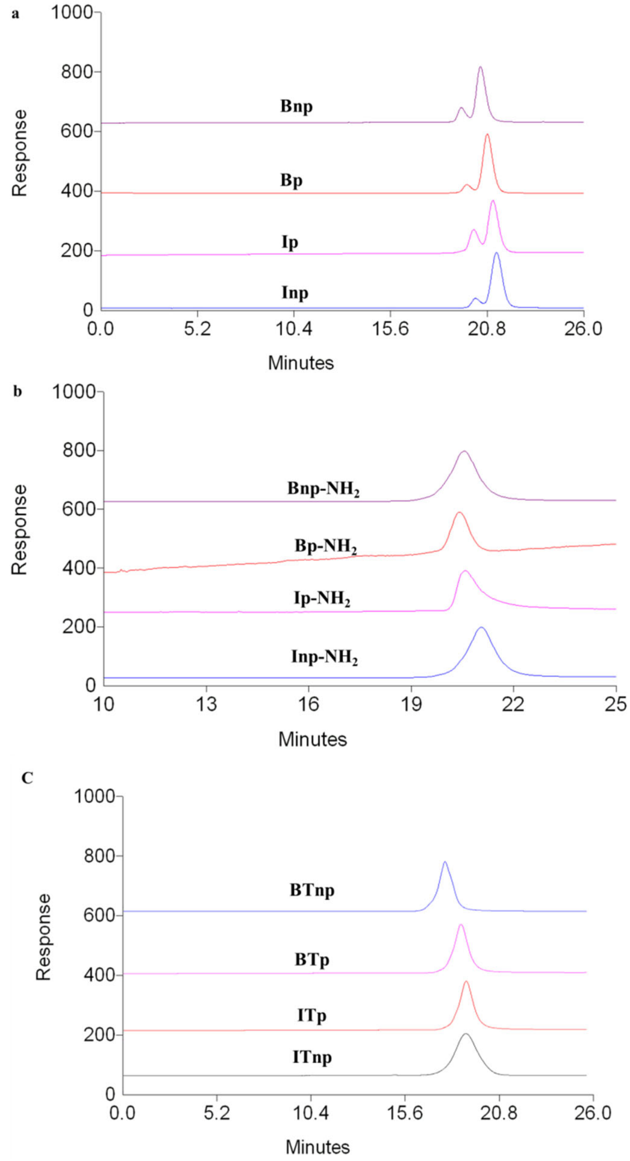

Figure S1: SEC chromatographs corresponding to: a. polydiene precursors, b. functionalized intermediate products and c. all final biobased hybrid copolymers.

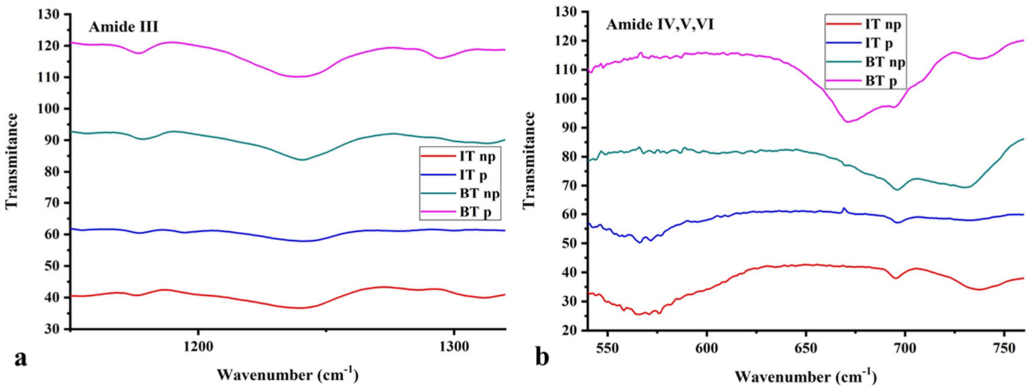

Figure S2: Magnified spectra corresponding to amide: a. III and b. IV, V, VI.

Table S1: Characteristic FT-IR peak wavenumbers corresponding to all components of the synthesized hybrid materials.

Chemical group	Range (cm^{-1})	ITnp	ITp	BTnp	BTp
**Amide VI/V/IV**	537-606	S	S	W	W
	640-800	M	M	S	S
	625-767	M	M	S	S
**Amide III**	1229-1301	M	M	S	S
**Amide II**	1545-1540 (α-helices)	-	-	-	-
	1530-1520 (β-sheets)	W	W	W	W
**Amide I**	1654-1667 (α-helices)	M	-	W	-
	1623-1643 (β-sheets)	M	S	W	S
**C=O**	1690-1760	✓	✓	✓	✓
**C-N**	1180-1360	✓	✓	✓	✓
**C=C (Ar)**	1500, 1600	✓	✓	✓	✓
**Ar-O-CH_2**	1000-1050, 1600-1650	✓	✓	✓	✓
**-NH_2**	1050-1250, >3000	✓	✓	✓	✓
**-NH-**	1050-1250, >3000	✓	✓	✓	✓

*S: strong, M: medium, W: weak, -: no existence and ✓: existence.

Table S2: Characteristic ^{1}H-NMR chemical shifts corresponding to all components of the synthesized hybrid materials.

Chemical group	Chemical shift (ppm)
**sec-BuLi/PB/PI Diaminohexane**	1.00-3.00
**$PI_{1,4}$**	1.80,2.85 & 5.44
**$PI_{3,4/1,2}$**	1.20-1.80, 4.90-5.70
**$PB_{1,4}$**	2.00, 2.85 & 5.70
**$PB_{1,2}$**	1.50, 3.00, 5.20 & 5.90
**-NH- (main chain linkage)**	>8.00
**-C^{a}H- /-NH_2 (end group)**	3.80 & 5.10
**P(o-Bn-L-tyr) (R group)**	3.30 & 5.20
**P(o-Bn-L-tyr) (R group: aromatic)**	6.90-7.50

Table S3: Characteristic ^{13}C-NMR chemical shifts corresponding to all components of the synthesized hybrid materials.

Chemical group	Chemical shift (ppm)
**$PI_{1,4}$**	16,32,47,124,140
**$PI_{3,4/1,2}$**	30,42,114,147
**$PB_{1,4}$**	34,38,127,131
**$PB_{1,2}$**	40-42,115,142
R: -$\underline{C}H_2$-ph-O-CH_2-ph	~40
**-C^{a}-**	~55
R: -CH_2-ph-O-$\underline{C}H_2$-ph	~70
**Aromatic**	114-130
**Aromatic (quaternary)**	130-160
**>$\underline{C}$=O ITnp & BTnp**	*167,172
**>$\underline{C}$=O ITp & BTp**	*174,172

*The first chemical shift is referred to the linkage between polydiene segment and the diaminohexane. The second chemical shift is referred to the polypeptide chain and was found to be the similar for all samples.

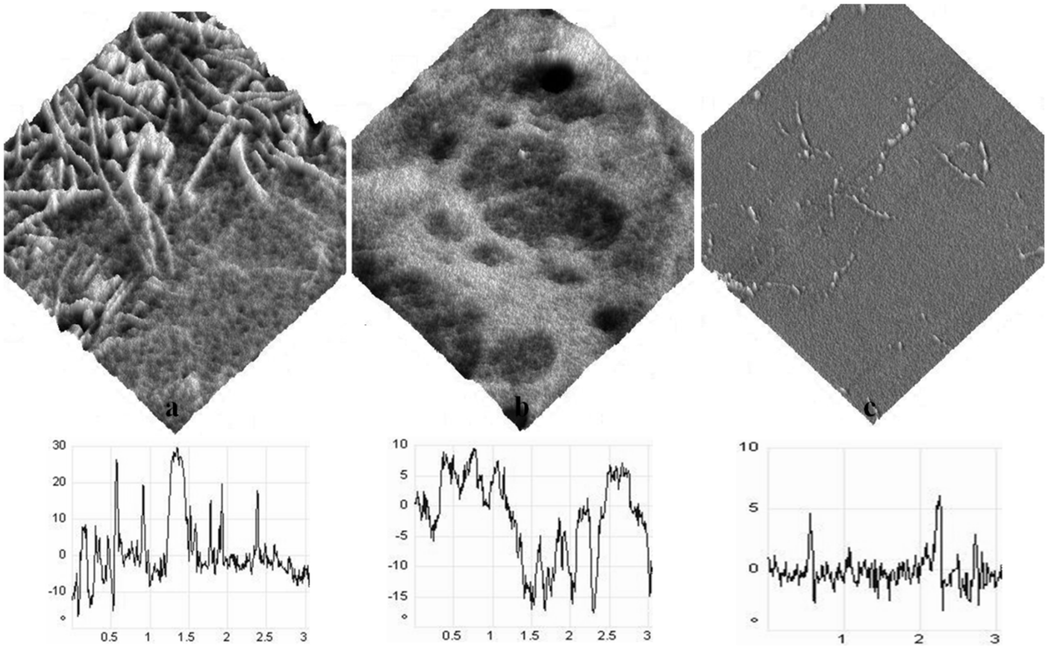

Figure S3: 3D AFM images (3 μm x 3 μm) at room temperature and the corresponding characteristic surface roughness profiles of a. $PB_{1,4}$-*b*-P(o-Bn-L-Tyr), b. $PB_{1,2}$-*b*-P(o-Bn-L-Tyr) and c. $PI_{1,4}$-*b*-P(o-Bn-L-Tyr).
